# Fe_3_O_4_/PMMA with Well-Arranged Structures Synthesized through Magnetic Field-Assisted Atom Transfer Radical Polymerization

**DOI:** 10.3390/polym16030353

**Published:** 2024-01-28

**Authors:** Ming Gao, Chi-Fai Cheung

**Affiliations:** State Key Laboratory of Ultra-Precision Machining Technology, Department of Industrial and Systems Engineering, The Hong Kong Polytechnic University, Hong Kong, China; benny.cheung@polyu.edu.hk

**Keywords:** magnetic composites, magnetic-field assisted polymerization, micro-structure, atom transfer radical polymerization, Fe_3_O_4_/PMMA

## Abstract

Particle- or fiber-reinforced polymer composites with controlled orientations are attracting interest and applications producing innovative materials, biological constructs, and energy devices. To gain the controlled orientations, filed-assisted synthesis is widely selected for its easy operation and control. In this paper, we designed magnetic field-assisted equipment and synthesized a magnetic polymer composite Fe_3_O_4_/PMMA with a well-arranged layers structure by combining the magnetic field with atom transfer radical polymerization (ATRP). During the polymerization of polymer composites, the magnetic nanoparticles were surrounded by monomers. With the growth of polymer chains, the magnetic particles pushed polymer chains to move according to a specific direction and form a well-arranged structure under the magnetic fields. The existence of a well-arranged layered structure of the composites gives potential guidance for controlling the micro-structure by adding an extra field during the polymerization process. The experimental results provided a possible design to influence the macroscale properties through control of the micro-structure of polymer composites.

## 1. Introduction

Over the past decades, the range of applied material selections has been wider because of the addition of reinforcements to polymer composite systems. The inorganic nanoparticles dispersed in polymeric matrices could improve the characteristics of polymer matrices, such as optical, thermal, mechanical, and magnetic properties. And the improvement is mainly decided by the properties of nanoparticles in the system [1]. Because of polymers’ low viscosity and melting temperature, the reinforcements have a broad range of uses with various shapes, properties, and sizes. Both fiber and particle reinforcements enhance the properties of the polymer matrix or significantly introduce new functionalities into the polymer matrix [2]. The primary processing of composites includes injection molding, filament winding, hand lay-up, bulk molding, resin transfer molding, etc. [3]. These processes could be divided into two classes, considering the nature of the composites. In the first class, the additives are addressed independently, and the polymer matrix works like a simple medium in which the additives are dispersed. Composites synthesized through these techniques usually control the alignment of additives by external forces based on the properties of fillers. The second kind of polymer composites synthesized based on the interaction between additives and polymeric matrix, and the interactions between molecular guides the formation of designed arrangement. Extra field assisted process could be used to synthesize polymer composites combined with polymerization process. In the field of polymerization, atom transfer radical polymerization (ATRP) is a widely used and versatile strategy that was developed by Matyjaszewski and colleagues in 1990s [4]. The ATRP technique allows for the precision and controlled synthesis of polymers containing characteristics of narrow molecular weight distributions, specific architectures, and predetermined molecular weights. This unique polymerization methodology brings a revolution to this field, enabling the polymerization of functional polymers with designed properties for different applications [5]. ATRP has a controlled radical polymerization mechanism, in which a transition metal complex catalyst works as a mediation of the halogen atoms exchanging process between initiators and growing polymer chains. The controlled propagation of polymer chains relies on the equilibrium between active species and dormant, which could be turned by controlling the concentration of the copper catalyst and the initiator [6,7]. This controlled property of ATRP gives it reasonable control of polymer architecture and makes the synthesis of well-defined structures like block copolymers, brush polymers, and grafted polymers easier [7,8,9]. The wide applications of ATRP rely on its ability to gain precise control over polymerization, resulting in polymers with well-defined characteristics and molecular weights. This nature of ATRP enables the turning of chemical, physical, and biological properties of materials, making ATRP an essential technique in designing advanced materials. However, despite its advantages, ATRP still faces challenges, such as difficulties in reducing agents and removing residual catalysts from the final product and the narrow scope of monomer selection [10]. 

One of the challenges in synthesizing polymer composites is forming a homogeneous mixture during the production process [11]. For particle-reinforced composites, the rapid coagulation of additives caused by their Van der Waals force, high surface energy, and capillary particles after mixing with polymer resin influence the final properties of composites [12,13]. In addition to these challenges, the micro-structure of polymer composites also dramatically affects the macro-properties of polymer composites. To overcome the above-mentioned problems, field-assisted additive manufacturing should be considered to synthesize polymer composites, and the manufacturing process has been used in some additive manufacturing processes, which is usually used by polymer composite materials. The history of field-assisted additive manufacturing (AM) could be traced back to the 1980s, when the concept of additive manufacturing was proposed. Additive manufacturing has been considered as a great breaking technology, which achieves the synthesis of complicated and tailored products with great design freedom. However, except for its potential, additive manufacturing often obtains challenges among limited speeds, material selected limitations, and accuracy. To solve these problems, external fields integrated into additive manufacturing systems have begun to attract researchers’ attention [13,14]. The crucial purpose of field-assisted AM technologies is to control and separate individual additives’ orientation together with position in a polymer resin to obtain minute-adjustment properties of the final products [15,16,17]. By providing an additive field, the control of materials flow is more precise, the speed of additive manufacturing techniques is faster [18,19], and the compatibility of materials is wider [20]. Magnetic field-assisted AM is one of the most promising techniques among field-assisted AM technologies. With the support of an additive magnetic field, the materials’ orientation and alignment could be controlled well. 

Polymer nanocomposites consist of nanoscale additives, and polymeric matrices are a crucial part of materials, which has previously been explained. In order to fully utilize the characteristics of nanoparticles, it is critical to manage the additives’ distribution in the polymer composites system. The reason is that the structural arrangement has an influence on the interaction between the two components. Precisely, the generation of new techniques and manufacturing processes to control and create ordered arrays is a significant under-researched field. The reason is that nanoscale ordering could provide unique functionality that cannot currently be realized in random composites [21]. Magnetic composites with a well-arranged structure have a wide range of applications. In the past few years, researchers have synthesized different polymer composites with various structures. For example, Lei Wang had synthesized Ti_3_C_2_Tx@Fe_3_O_4_/CNF aerogels (BTFCA)/epoxy with long-range aligned lamellar structures, which work as electromagnetic interference (EMI). The designed structure increases the EMI shielding effectiveness and reduces the secondary contamination [22]. The EMI shielding property enables the material to be used in fifth-generation (5G) mobile communication technology [23]. Magnetic particles in a composite system could be used to turn the functions of the materials by influencing and controlling the microstructure of the materials. For example, researchers synthesized Fe_2_O_3_@Au core-shell nanoparticles to turn the pKa of 11-mercaptoundecanoic acid by containing the thickness of the shell [24]. Due to the advantages of magnetic polymer composites, such as bio-friendly, reusability, superparamagnetism, and the potential to turn the well-arranged microstructure, it has attracted increasing attention. In this project, Fe_3_O_4_ nanoparticles in the system were successfully used as a tool to guide and influence the molecular flow and further influence the arrangement of the microstructure of the synthesized polymer composites. The results of this experiment provide a potential for turning the macroscale characteristics by controlling the microstructure of the materials with the assistance of an extra applied field. 

PMMA is one of the most widely used industrial polymer materials because of its mouldability, biocompatibility [25], extraordinary optical transparency [26], mechanical properties, and weatherability [27]. Several nanoparticles/PMMA composites were synthesized through the ATRP technique in the past few decades to enhance or add various properties to PMMA [28,29,30]. In this paper, compared with the typical synthesized process of magnetic polymer composites, a magnetic field-assisted ATRP technique was a novelty used to synthesize homogeneous magnetic polymer composites Fe_3_O_4_/PMMA containing well-arranged micro-structure. Compared with other methods of particle manipulation, magnetic field-assist technology has shown more flexibility and is more straightforward to use [31,32]. 

**Figure 1:**
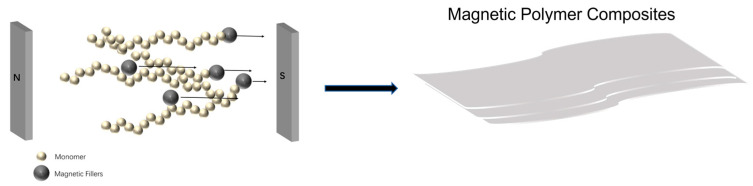
Graphic scheme of the project.

The scheme of this paper is shown in Figure 1 below. Generally, the polymerization was assisted by an extra magnetic field. During the polymerization, the magnetic particles in the system draw and push the polymer chains to move according to the direction of the magnetic field. As a result, a well-arranged structure could be synthesized. 

## 2. Materials 

Amounts of 99% Methyl methacrylate (MMA), 98% Copper (I) bromide, 99% Copper (II) bromide, 99.9% N,N-Dimethylformamide (DMF), 98% 1,1,4,7,7-Pentamethyldiethylenetriamine were purchased from FineLab Scientific Limited (Hong Kong, China). Additionally, 98% α-Bromoisobutyryl bromide, 99% Butanol, AR 99.5% acetic acid, 98% L-Ascorbic acid, and 99.5% Ferrosoferric oxide (20 nm) were purchased from TiV Scientific Limited (Hong Kong). 

**Figure 2:**
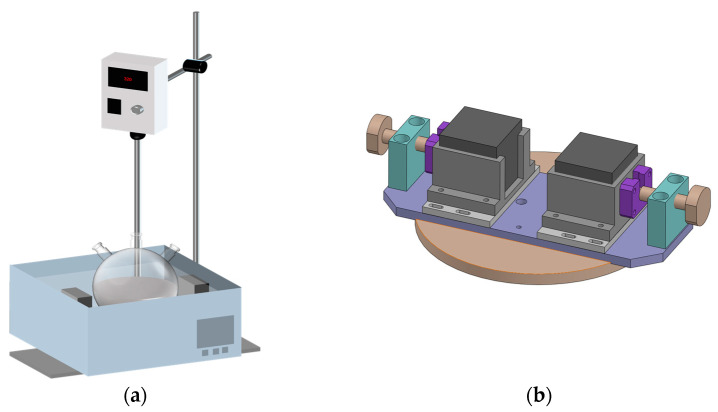
Schemes figures: (**a**) whole designed reactor; (**b**) magnetic field assistant system.

A magnetic synthesized system should be designed to synthesize magnetic polymer composites under a magnetic field through ATRP. The schematic diagrams of the synthesized system are listed in Figure 2. The whole system contains three parts. The first session is the 100 mL two-neck spherical reaction flask connected to a glass double manifold vacuum gas distributor in which the polymerization process occurred without oxygen. The second part is a tunable magnetic field system, and the magnets are located on the two opposite sides of the system. The strength of the magnetic field could be turned by adjusting the distance between the two magnets, which is shown in Figure 2b. The third part is the oil bath with a mechanical stirrer, which is used to control the temperature of the whole reaction. 

## 3. Experiments

**Figure 3:**
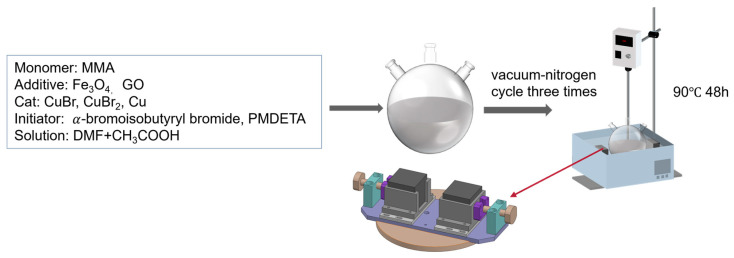
Schematic diagram of magnetic field-assisted ATRP experiment.

Generally, Fe_3_O_4_ was dispersed into N,N-Dimethylformamide (DMF) by an ultrasonic process to form a uniform solution. The solution containing methyl methacrylate (MMA), catalyst, and initiator of the ATRP reaction was added into the system. Through the ATRP reaction, the magnetic polymer composite, Fe_3_O_4_/PMMA, was fabricated. The schematic diagram is shown in Figure 3 below.

I. Purification of catalyst

Firstly, pretreatment of CuBr was needed. CuBr was dissolved into enough CH_3_COOH by magnetic stirring at room temperature overnight to remove impurities in CuBr. Next, methyl alcohol was used to wash the purified CuBr 3 times, and the gained CuBr was placed in a vacuum oven and dried for 12 h at 40 °C. After drying, the purified CuBr was kept under vacuum in a desiccator.

II. Polymerization of Fe_3_O_4_/PMMA

Different weight concentrations of Fe_3_O_4_ (0.35 wt%, 0.7 wt%, 2.5 wt%, 5 wt%, 10 wt%, 20 wt%, which are 0.065 g, 0.130 g, 0.511 g, 0.93 g, 1.80 g, 3.76 g, respectively) were dispersed into 15 mL of DMF by an ultrasonic process for 30 min. The solution was kept as solution A. Then, 0.152 g of CuBr, 0.088 g of CuBr_2_, and 0.036 g of L-ascorbic acid were dissolved in a mixed solution consisting of 10 mL of DMF and 4 mL of CH_3_COOH by mechanical stirring. The dissolving process proceeded in a 100 mL two-neck spherical reaction flask, and the solution was kept as solution B. Subsequently, 20 mL of MMA and solution A, with a particular concentration of Fe_3_O_4_, were added into solution B, and the mixed solution was well dispersed through mechanical stirring to form solution AB. Three vacuum–nitrogen cycles were purged into the system to remove the oxygen in solution AB before starting the ATRP process. 

Following this, 80 μL of α-bromoisobutyryl bromide and 142.4 μL of 1,1,4,7,7-Pentamethyldiethylenetriamine (PMDETA) were added into the solution AB in a two-neck spherical reaction flask by dropping through an injection syringe. Then, the apparatus was heated to 90 °C and bathed in oil for 48 h to facilitate the reaction under a 200 mT magnetic field. The reactants were added according to a molar ratio of 287[MMA]:1[-Br]: 324[CH_3_COOH]:2[PMDETA].

III. Polymerization of pure PMMA

A similar process synthesized pristine PMMA. Firstly, 10 mL of DMF and 4 mL of CH_3_COOH were mixed together to form a solution A. Then, 0.152 g of CuBr, 0.088 g of CuBr_2_, and 0.036 g of L-Ascorbic acid were added and stirred well to obtain a homogeneous solution B, followed by adding 20 mL of MMA and solution to form a homogeneous solution AB by mechanical stirring. To remove oxygen in the system, three vacuum–nitrogen cycles were taken before the polymerization. 

Next, 80 μL of α-bromoisobutyryl bromide and 142.4 μL of PMDETA were injected into the solution AB in a two-neck spherical reaction flask by a syringe. Then, polymerization was triggered by heating the apparatus to 90 °C and keeping it in an oil bath for 48 h. The reaction system schematic diagrams are drawn in Figure 3.

IV. Collection of synthesized samples

After polymerization, the synthesized polymer composites were collected through a vacuum filter. The products were washed three times through the dissolving and filtering process. The purified products were then dried overnight at 80 °C. After drying, a hot press process was taken to gain the polymer sheet at 145 °C, followed by a pressing process at 150 °C with 30 KN to further deform the sheet into circular disks with 50 mm diameter and 1 mm thickness.

V. Testing techniques and conditions

The morphologies of obtained samples were studied by a scanning electron microscope (TESCAN VEGA3) and field emission transmission electron microscope (JEOL JEM-2100F)). The samples were learned through Fourier-transform infrared spectroscopy (FTIR) spectrometers (Perkin Elmer) with a wavenumber ranging from 500 to 4000 cm^−1^. X-ray diffraction (XRD, Rigaku Smartlab, Tokyo, Japan) was used to study the crystallization structures scanning from 15° to 85°. Differential scanning calorimetry (DSC, METTLER 1100LF) was used to learn the thermal properties, and the temperature range was from 30 °C to 140 °C in Ar flow with a temperature rising rate of 10 °C/min. The molecular weight and distribution of the polymer chain were detected by a Shimadzu Rid-20A (Kyoto, Japan), and the polymer was dissolved in tetrahydrofuran (THF) to make a solution of 20 mg/mL. This study used Zygo Nexview Optical Surface Profiler to test the surface roughness (Sa) of Fe_3_O_4_/PMMA samples with different amounts of Fe_3_O_4_. All the samples were machined at a feed rate of 400 mm/min and 3 μm of cut.

## 4. Results and Discussion

**Figure 4:**
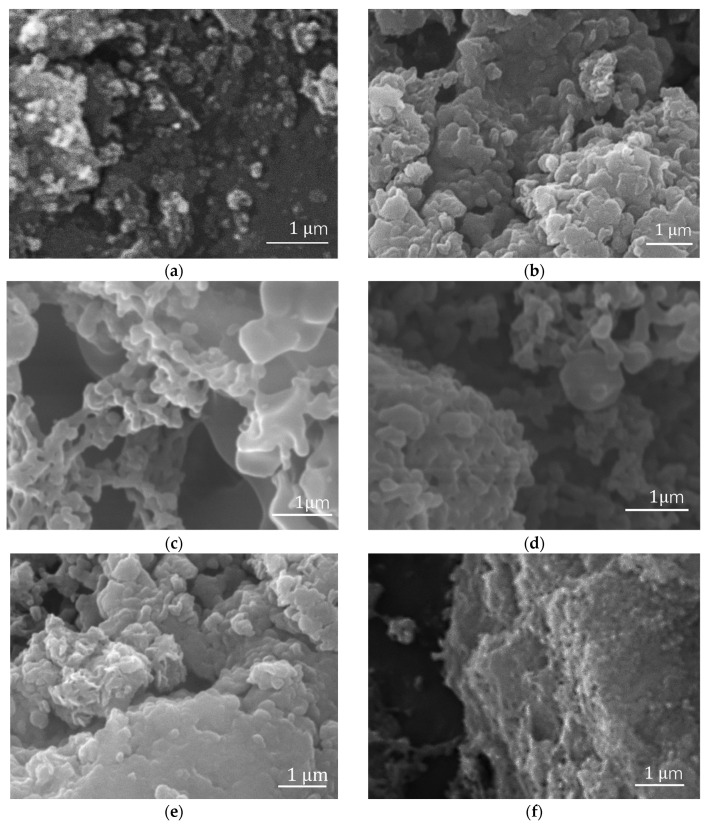

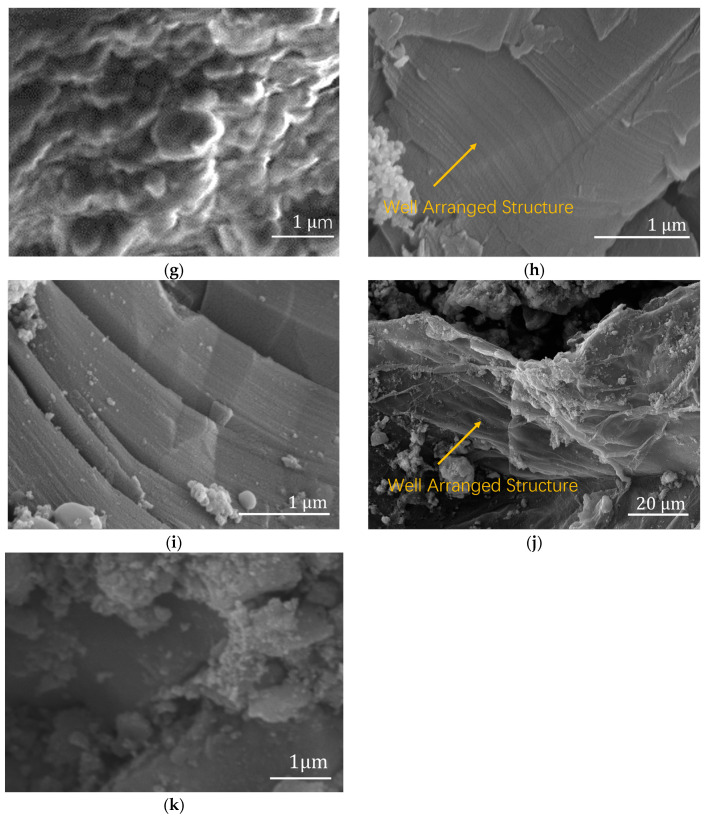
SEM pictures: (**a**) Fe_3_O_4_; (**b**) PMMA; (**c**) Fe_3_O_4_/PMMA (0.35 wt%); (**d**) Fe_3_O_4_/PMMA (0.7 wt%); (**e**) Fe_3_O_4_/PMMA (2.5 wt%); (**f**) Fe_3_O_4_/PMMA (5 wt%); (**g**) Fe_3_O_4_/PMMA (10 wt%); (**h**–**j**) Fe_3_O_4_/PMMA (20 wt%); (**k**) Fe_3_O_4_/PMMA (20 wt%) synthesized without magnetic field.

The morphology and structure of each reaction product were observed by a TESCAN VEGA3 scanning electron microscope and a transmission electron microscope. Figure 4a shows the morphology of Fe_3_O_4_ nanoparticles. Because of their magnetic properties, these nanoparticles have a solid intermolecular force to reunite. After ultrasonic mixing, Fe_3_O_4_ particles were dispersed. After Fe_3_O_4_ is added to the ATRP system, polymer chains grow under a magnetic field, with the magnetic fillers affecting the chains’ arrangement. 

Compared with the morphology of pure PMMA in Figure 4b and Fe_3_O_4_/PMMA (20 wt%) synthesized without magnetic field, Figure 4h–j show the obvious oriented structure of the polymer composite with 20 wt% weight concentration of magnetic additives Fe_3_O_4_. When the weight concentration of magnetic fillers increases from 0.35 wt% to 20 wt%, the arrangement of polymer chains grows from a small plate structure to a well-arranged structure. Compared with polymer composites with 10 wt% weight concentrations of magnetic additives, the well-arranged structure is more apparent when the weight concentration grows to 20 wt%. When monomers begin to grow, the small chains are separated and guided by the magnetic nanoparticles in the system under a specific magnetic field of 200 mT. The direction of polymer chains’ growth is assisted by the combination of magnetic fillers and magnetic fields. As a result, when the concentration of magnetic fillers increases to 20 wt%, a large, well-arranged PMMA layered structure is formed, as shown in Figure 4f–h.

**Figure 5:**
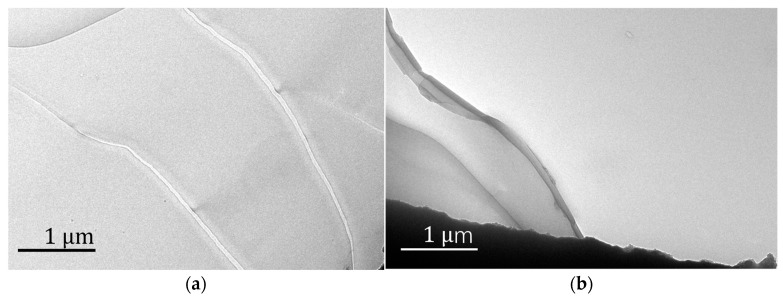
TEM pictures of Fe_3_O_4_/PMMA (20 wt%): (**a**) surface; (**b**) layered structure.

Figure 5 shows the TEM pictures of the well-arranged structure of Fe_3_O_4_/PMMA (20 wt%). The TEM results clearly show the well-arranged layered structure of polymer composites, which confirms the functions of added magnetic nanoparticles and magnetic field. Under the magnetic field, magnetics, Fe_3_O_4_ particles have an influence on the structure of polymer composites during the polymerization process.

**Figure 6:**
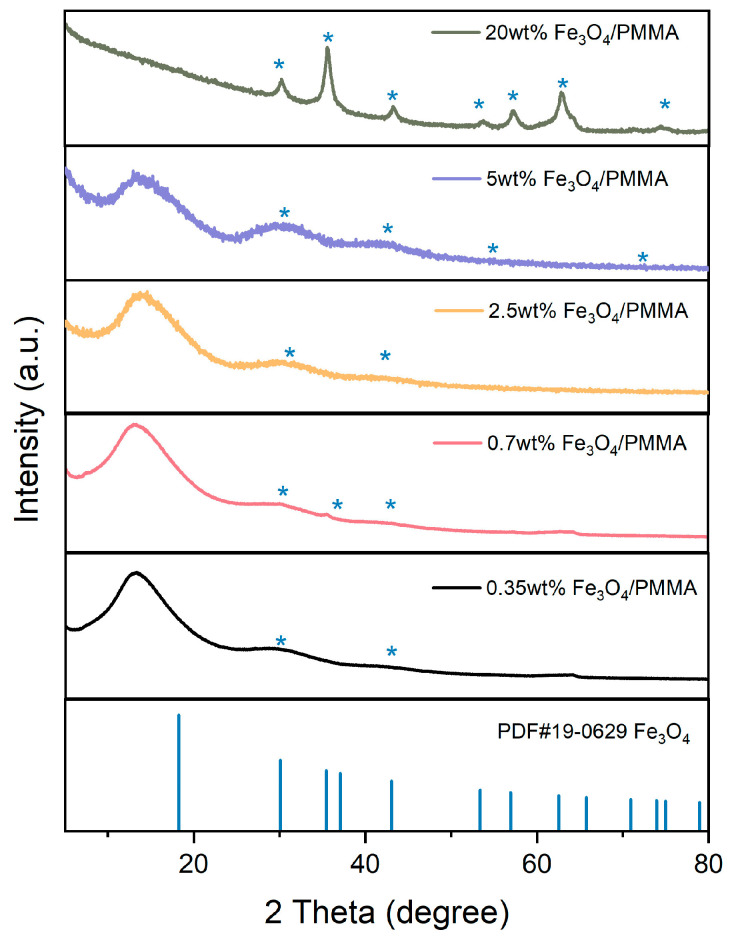
XRD results of synthesized products with various weight percentages of additives.

The crystallization structures of products were analyzed by X-ray diffraction. The spectra of synthesized composites, Fe_3_O_4_/PMMA, with different weight percentages of magnetic additives Fe_3_O_4_, are detected from 15° to 85° and shown in Figure 6. 

The PDF card of Fe_3_O_4_PDF#19-0629 is represented by a blue line. Peaks of 30°, 35° 53°, 56°, 71°, and 73° represent Fe_3_O_4_, and it could be found that these typical peaks are observed in XRD patterns of Fe_3_O_4_/PMMA composites; with the addition of magnetic fillers, peaks of Fe_3_O_4_ are more significant. When the concentration of Fe_3_O_4_ is added to 20 wt%, the XRD spectra of polymer composites show clear and significant typical peaks of Fe_3_O_4_. 

**Figure 7:**
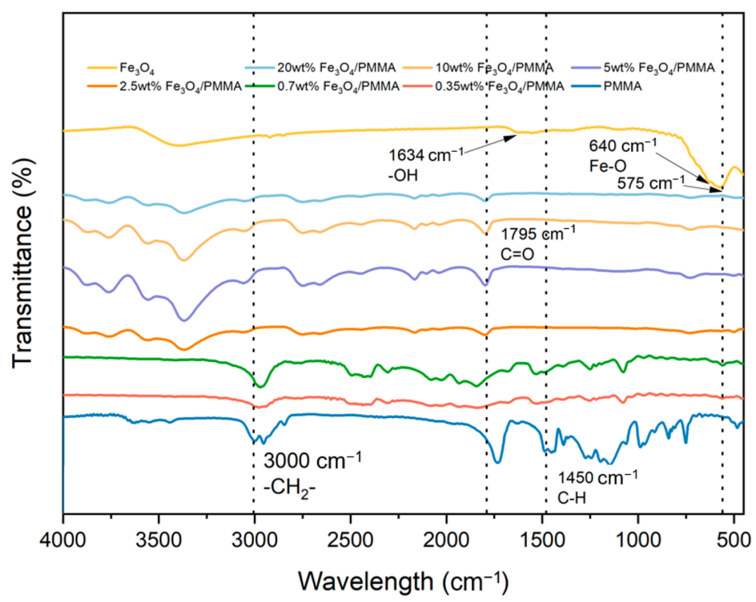
FTIR spectra of polymer composites with different weight percent of additives.

A Perkin Elmer Fourier-transform infrared spectrometer was adopted to study the polymer structure with a wavenumber ranging from 500 to 4000 cm^−1^. Figure 7 shows the measured results for Fe_3_O_4_ and Fe_3_O_4_/PMMA with different weight percentages of Fe_3_O_4_ and pure PMMA.

The two intense peaks with small wavenumbers at 575 cm^−1^ and 640 cm^−1^ represent the stretching vibration mode of Fe-O metal–oxygen bonds in the Fe_3_O_4_ crystalline lattice. These two peaks are also found in polymer composites, which proves the successful connection between polymer resin and Fe_3_O_4_ additives. The typical peaks of PMMA can also be observed in Figure 5. Peaks at 1450 cm^−1^, 1634 cm^−1^, 1765 cm^−1^, and 3000 cm^−1^ represent C-H, -OH, C=O, and -CH2- of PMMA, respectively. The FTIR spectra of Fe_3_O_4_/PMMA show clear peaks of PMMA and Fe_3_O_4_. Combined with the results from XRD, the successful synthesis of Fe_3_O_4_/PMMA could be proven. 

**Figure 8:**
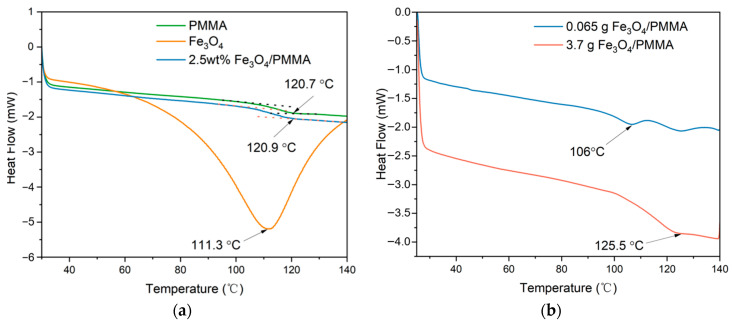
DSC results: (**a**) PMMA, 2.5wt% Fe_3_O_4_/PMMA, Fe_3_O_4_; (**b**) magnetic polymer composites with different concentrations of Fe_3_O_4_ (0.3 wt%, 20wt%).

Differential scanning calorimetry (DSC) was tested to observe the thermal characteristics of different composites, and the results are shown in Figure 8 below. The tested temperature ranges from 30 °C to 140 °C at an increment of 10 °C /min under N_2_ atmosphere. The DSC results of PMMA, Fe_3_O_4_, 0.35 wt% of Fe_3_O_4_/PMMA, 2.5 wt% of Fe_3_O_4_/PMMA, and 20 wt% of Fe_3_O_4_/PMMA are shown in Figure 8. From the DSC results, it can be found that when the concentration of magnetic fillers is low, the glass-transition temperature (T_g_) decreases considerably from 120.7 °C to 106 °C compared with pure PMMA. When the concentration of magnetic fillers increases to 2.5 wt%, the T_g_ of Fe_3_O_4_/PMMA rises to 120.9 °C, which is close to the T_g_ of pure PMMA. The decrease in T_g_ with low weight concentration of magnetic additives could be explained by the fact that nanoparticles between the polymer chains decrease the intermolecular force between macromolecules.

With the further increase in the concentration of Fe_3_O_4_ to 20 wt%, the T_g_ of Fe_3_O_4_/PMMA rises to 125.5 °C. The growth of T_g_, when the concentration of magnetic fillers becomes higher, may be caused by the restrictions of polymer chains’ movement by rigid Fe_3_O_4_ nanoparticles inside the polymer composites. The larger attractive forces between polymer chains from magnetic particles also lead to the higher T_g_ of polymer composites. The application of an extra magnetic field is to control nanoscale organization. In this experiment, magnetic fields could help to arrange the alignment of magnetically responsive Fe_3_O_4_ nanoparticles. Combined with SEM and TEM results, it could be found that the polymer composites have a well-arranged microstructure when the weight percentage of additives reached 20 wt%. With the well-ordered microstructure, the interaction between polymer chains makes the movement of polymer chains harder, which increases the T_g_ of the polymer matrix. Although the differences between pure PMMA and polymer composites are insignificant, this observation illuminates a potential strategy to modify composite characteristics by controlling micro-organization within the materials.

**Figure 9:**
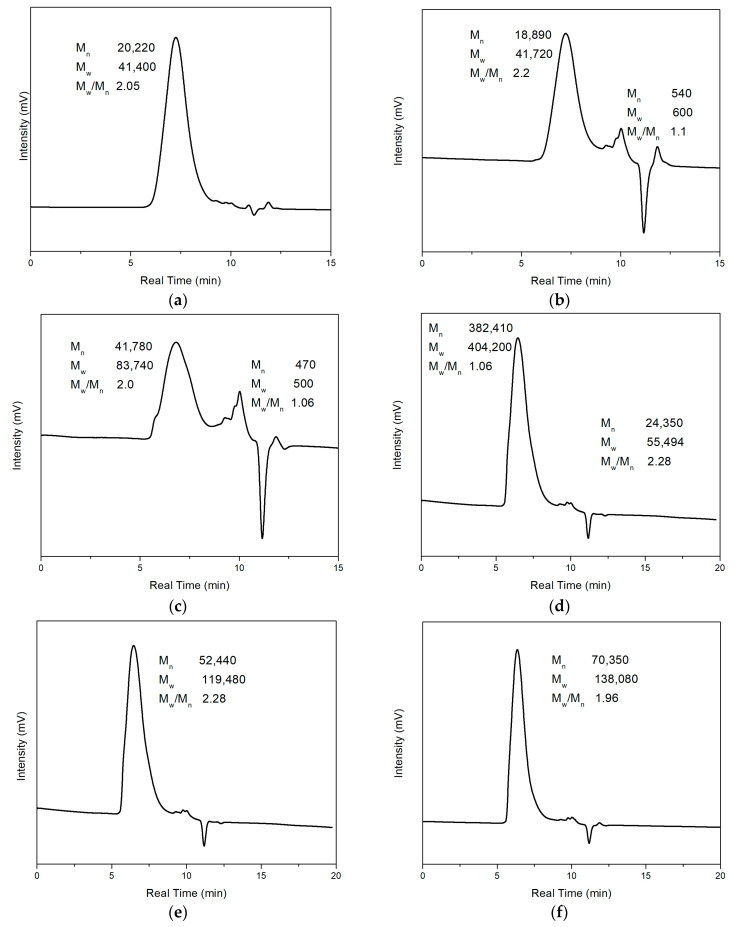

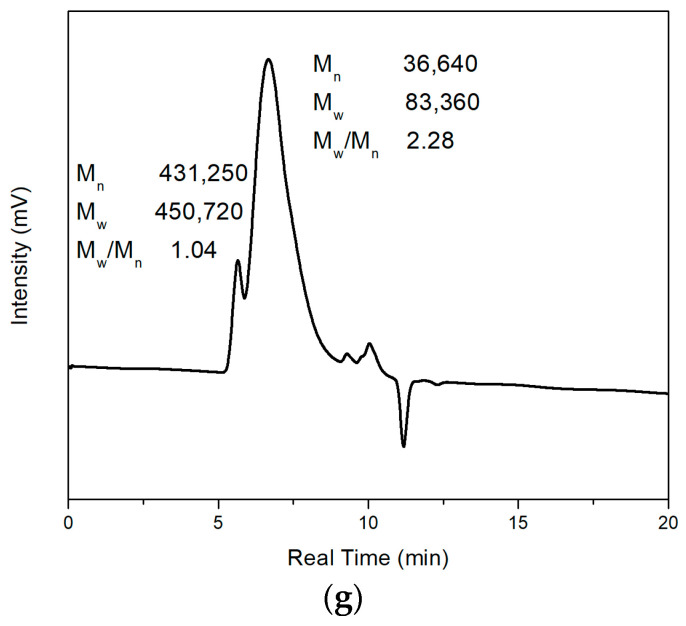
GPC results: (**a**) PMMA; (**b**–**g**) Fe_3_O_4_/PMMA with different concentration of Fe_3_O_4_ particles (0.35 wt%, 0.7 wt%, 2.5 wt%, 5 wt%, 10 wt%, 20 wt%, respectively).

The molecular weight of PMMA and Fe_3_O_4_/PMMA with varying Fe_3_O_4_ weight percentages was measured by GPC. The testing solution was prepared by dissolving the products into tetrahydrofuran (THF), forming a 20 mg/mL concentration solution, and then the results were tested by a Shimadzu Rid-20A (Kyoto, Japan) machine. Figure 9a shows the GPC result of pure PMMA, and Figure 9b–g shows the GPC results of synthesized composites with different weight percentages of Fe_3_O_4_ particles. Figure 9b,c shows two significant peaks among these figures. The left peak represents larger-molecular-weight polymer chains, and the right peak represents molecules with small molecular weights of around 500.

Figure 9a shows the GPC results of pure PMMA. The number average molecular weight (M_n_) and weight average molecular weight (M_w_) of PMMA are 20,220 and 41,400, respectively, and M_w_/M_n_ = 2.2 could calculate the dispersity of pure PMMA. Figure 9b–g shows the polymer composites with different concentrations of magnetic particles (0.35 wt%, 0.7 wt%, 2.5 wt%, 5 wt%, 10 wt%, 20 wt%, respectively). The M_n_ of magnetic polymer composites is 18,890, 41,780, 382,410, 52,440, 70,350, and 36,640, respectively, and the M_w_ is 41,720, 83,740, 404,200, 119,480, 138,080, and 83,360, respectively. It could be found that except for Fe_3_O_4_/PMMA with 0.35 wt% Fe_3_O_4_ additives, other composites have larger M_n_ compared with pure PMMA. The larger molecular weight may come from adding an external magnetic field. Under the magnetic field direction, the flow of molecules was affected, and the unified direction of molecular flow led to easier elongation of polymer chains. However, when the concentration of magnetic additives was insufficient, the influence from the magnetic field was not significant, which led to the similar molecular weight of pure PMMA and Fe_3_O_4_/PMMA (0.35 wt%). The GPC results have been concluded and shown in Table 1 below. The large dispersity may be because of the long polymerization time. With longer polymerization time, the polymerization period before termination of polymer chains may be significantly different, leading to a large dispersity of molecular weight. 

**Figure 10:**
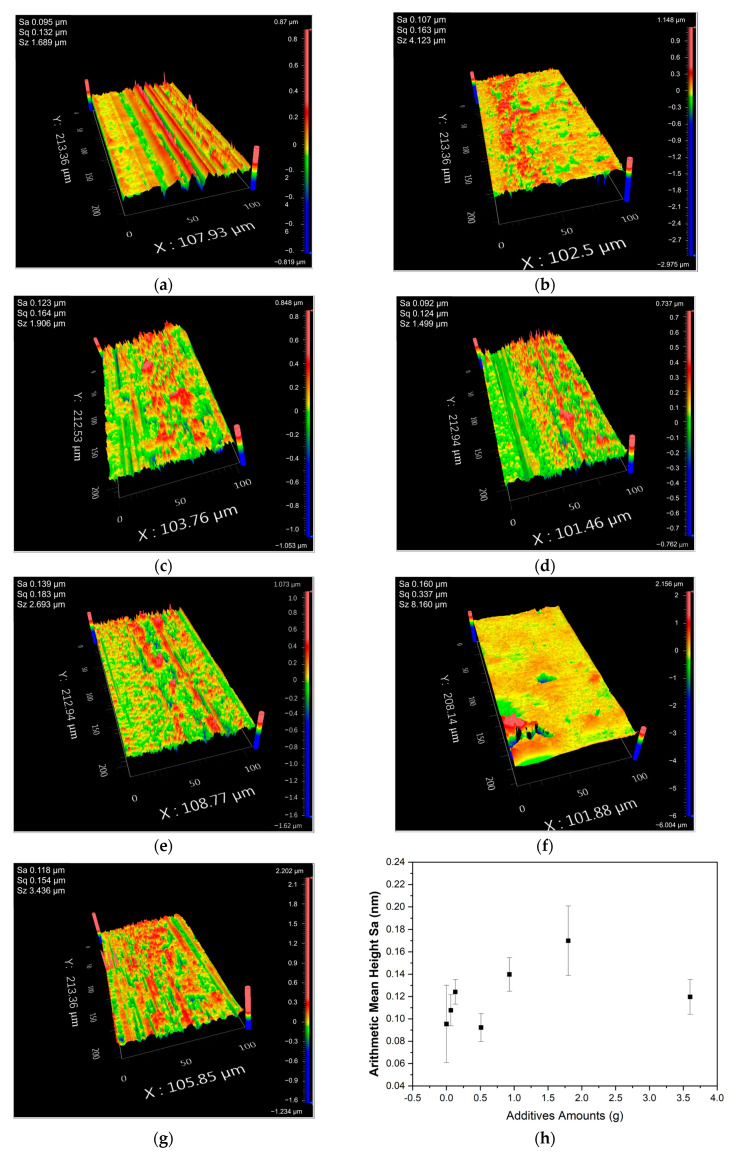
Surface integrity of pure PMMA and Fe_3_O_4_/PMMA with different amounts of Fe_3_O_4_ after machining: (**a**) pure PMMA; (**b**) Fe_3_O_4_/PMMA (0.35 wt% of Fe_3_O_4_); (**c**) Fe_3_O_4_/PMMA (0.7 wt% of Fe_3_O_4_); (**d**) Fe_3_O_4_/PMMA (2.5 wt% of Fe_3_O_4_); (**e**) Fe_3_O_4_/PMMA (5 wt% of Fe_3_O_4_); (**f**) Fe_3_O_4_/PMMA (10 wt% of Fe_3_O_4_); (**g**) Fe_3_O_4_/PMMA (20 wt% of Fe_3_O_4_); (**h**) surface roughness in term of arithmetical mean height (Sa) results with varying weight percentage of additives.

This study used Zygo Nexview to test the surface roughness (S_a_) of Fe_3_O_4_/PMMA samples with different amounts of Fe_3_O_4_. All the samples were machined at a feed rate of 400 mm/min and 3 μm of cut. Figure 10 shows the surfaces of workplaces after machining. From Figure 10a–g, the concentrations of additives are 0 wt%, 0.35 wt%, 0.7 wt%, 2.5 wt%, 5 wt%, 10 wt%, and 20 wt%, respectively. 

The surface roughness in terms of arithmetical mean height (Sa) results with the error bar are summarized in Figure 10h. The results show that the resultant surface roughness is positively proportional to the additive amount, despite an exceptional case found in 2.5 wt% Fe_3_O_4_. However, when the additive concentration reached 20 wt%, a significant decrease in surface roughness was found in the workplace. The additives in composites break the Van der Waals force between polymer chains, which causes an increase in brittleness. When the concentration of additives is small, the particles inside the composites work as impurities, decreasing the ductility and machinability. However, when the concentration of magnetic fillers increases to 20 wt%, the magnetic fillers surrounded by the polymer chains move according to the magnetic field’s direction. As a result, the polymer composites form a well-arranged structure with a more significant Van der Waals force, affecting the macro-properties of polymer composites. The well-arranged structure enhances the attraction between polymer chains, increasing the ductility and decreasing the brittleness of polymer composites, leading to better machinability. 

## 5. Conclusions

In this paper, magnetic polymer composites Fe_3_O_4_/PMMA with different concentrations of Fe_3_O_4_ were synthesized by magnetic field-assisted ATRP technology. This paper novelty combined an extra magnetic field with the ATRP process and tried to influence the macroscale characteristics by turning the micro-structure with the assistance of an extra field. With the addition of Fe_3_O_4_, the machinability of composites was decreased until the amount of Fe_3_O_4_ reached 20 wt%. The reason for the improvement of the machinability is the well-arranged layered microstructure of the synthesized polymer composites with 20 wt% of magnetic additives. The microstructure gives composites a stronger Van der Waals force between polymer chains, leading to an increase in ductility and improved machinability. Except for the machinability, the glass transition temperature (T_g_) of polymer composites also increased lightly from around 120 °C to 125 °C. The microstructure has an influence on macro-properties like ductility and thermal stability. Magnetic field-assisted technology provides a simple and easy-controlled method to control the microstructure of polymer composites. The results of this paper offer a potential strategy to influence and turn the macroscale characteristics through influence or control of the micro-structure of polymer composites with the assistance of extra field. The well-organized micro-structure can be further applied as a strategy to organize the alignment of other small molecules in the composite system so as to control the characteristics of the small molecules. 

## Figures and Tables

**Table 1:** GPC results of PMMA and Fe_3_O_4_/PMMA composites with different concentrations of Fe_3_O_4_ additives.

Concentration of Fe_3_O_4_	0 wt%	0.35 wt%	0.7 wt%	2.5 wt%	5 wt%	10 wt%	20 wt%
M_n_	20,220	18,890	41,780	382,410	52,440	70,350	36,640
M_w_	41,400	41,720	83,740	404,220	119,480	138,080	83,360
M_w_/M_n_	2.05	2.2	2.0	1.06	2.28	1.96	2.28

## Data Availability

Data are contained within the article the original contributions presented in the study are included in the article, further inquiries can be directed to the corresponding author/s.
